# Response to letter to editor from Rattanapitoon, N. & Rattanapitoon, S

**DOI:** 10.1017/S0950268825100459

**Published:** 2025-09-12

**Authors:** Sara R. Healy, Martha Betson, Joaquin M. Prada, Eric R. Morgan

**Affiliations:** 1 School of Veterinary Medicine, University of Surrey, Surrey, UK; 2 Institute for Global Food Security, Queen’s University, Belfast, UK

To the editor,

We would like to thank the authors of the correspondence for their thoughtful and generous engagement with our recent article [1]. We greatly appreciate their recognition of the modelling framework we developed to assess the foodborne risk of *Toxocara* spp. transmission in the UK context, and we are encouraged by their insightful reflections on its broader applications and future refinements.

We particularly welcome the emphasis on the influence of cultural dietary practices and regional variability in food preparation, which are indeed key factors in shaping exposure risk. As noted, the model was calibrated using UK-specific data, but the structure of the framework was deliberately designed to be adaptable to support comparative assessments across diverse settings. Where contamination levels vary among food types, for example, they will interact with local food choices and culinary practices to drive exposure risk [2, 3]. We also acknowledge the current uncertainty surrounding the thermal inactivation thresholds of *Toxocara* spp. larvae in meat tissues. Addressing this knowledge gap through further empirical research would provide valuable data for refining risk assessments and developing evidence-based consumer guidance – especially in settings where raw or lightly cooked meat is commonly consumed.

We also appreciate the comments regarding diagnostic specificity and the potential for improved granularity in distinguishing between *T. canis* and *T. cati* exposures. As more refined serological tools become available, future iterations of the model could incorporate such advances, enabling more nuanced interpretation of seroprevalence data [4].

In conclusion, we are very grateful for the correspondents’ thoughtful suggestions and support for this work. We share their view that the development and expansion of quantitative models for neglected foodborne parasitic risks is both timely and necessary. We hope our framework can serve as a foundation for future context-specific adaptations, advancing One Health surveillance and risk communication globally.

Yours sincerely,
